# Effect of early onset otitis media on brainstem and cortical auditory processing

**DOI:** 10.1186/1744-9081-4-17

**Published:** 2008-04-02

**Authors:** Sandeep Maruthy, Jayaram Mannarukrishnaiah

**Affiliations:** 1 Department of Audiology, All India Institute of Speech and Hearing, Mysore, India; 2 Department of Speech Pathology and Audiology, National Institute of Mental Health Neuro Sciences, Bangalore, India

## Abstract

**Background:**

Otitis media (OM) leads to significant reduction in the hearing sensitivity. The reduced auditory input, if in the early years of life when the auditory neural system is still maturing, may adversely influence the structural as well as functional development of the system. Past research has reported abnormalities in both the structure and function of brainstem nuclei following auditory deprivation, but, it has not necessarily focused on children who had OM in their first year of life. It can also be said that if auditory processing is affected at the brainstem level because of early onset OM (reduced auditory input in the crucial periods of neural development), then, it may be said that auditory processing is also affected at the cortical level because it receives distorted input from the brainstem. Therefore, the purpose of this study was to document the effects of early onset OM on auditory processing, if any, at the brainstem as well as at cortical levels. A related purpose of the study was to investigate the persistence of the effects of early onset OM, if any, on auditory processing.

**Methods:**

A cross sectional approach and a standard group comparison design was used in the study. Thirty children, who had OM between 6 and 12 months of age and who were in the age range of 3.1 – 5.6 years participated in the study. Children with OM were divided into 3 groups based on their age. Click evoked auditory brainstem responses (ABRs) and late latency responses (LLRs) were recorded from these children, and the responses were compared with those from age and gender matched normal children without any history of OM. The data from the 2 groups was statistically analyzed through independent *t *test. Pearson's Product Moment correlation was computed to examine the relationship between results of ABR and LLR in children with early onset OM.

**Results:**

The mean central conduction time was significantly increased and the mean amplitude of wave I and III of ABRs was significantly reduced in children with early onset OM compared to normal children. Also, the latency of all LLR waves was significantly less in children with early onset OM than in normal children. However, significant differences in mean values of either ABR or LLR (latencies or interwave intervals as the case may be) were observed only in 3-year old children. There was a significant, but negative association between central conduction time and latency of LLRs.

**Conclusion:**

OM in the first year of life leads to negative effects on brainstem signal processing even if it has occurred only for a short duration (maximum of 3 months). In such a situation, auditory cortical structures probably show compensatory changes through central gain to offset the prolonged central conduction time. Although the results of the present study showed that the negative effects of early onset OM (occurring in the first year of life) on auditory processing disappeared by the time the children were 4.1 years, there is need for longitudinal studies on this to confirm the findings.

## Background

Adequate sensory experience is critical to the developing nervous system – for the expression as well as maintenance of sensory functions even when such functions are innately determined [1]. Development and maintenance of auditory sense is no exception. In other words, reduced auditory input, early in life, may affect auditory processing later in life. Otitis media (OM) is a common condition that results in hearing loss in early years of life. Sandeep and Jayaram [2] have reported that OM occurring early in life may lead to subtle difficulties in speech identification, particularly under adverse listening conditions. Furthermore, such negative effects may persist for 4 years or even more after an attack of OM.

OM is the most prevalent disease during childhood, next only to common cold. It is estimated that chronic OM affects 65 million to 330 million people worldwide, and 60% of them (39 million to 200 million) show clinically significant hearing impairment [3]. Incidence of OM is known to be higher in the first 3 years of life [4]. Jayaram [5], in an Indian population, reported that OM was the cause of conductive hearing loss in nearly 71% of the 1505 persons ranging in age from 1 – 80 years. Similar results have been reported also by Parsram and Jalvi [6].

Past research has demonstrated that early OM in children influences auditory brainstem physiology [7]. Webster and Webster [7] reported a reduction in both the size and number of neurons in the auditory brainstem in subjects with OM. Past research has documented prolonged latencies for wave III and wave V [8-13], delayed wave III and prolonged III–V wave intervals [8], prolonged wave III–V interval [9] and, prolonged wave III–V and I–V intervals [10,11]. Anteby et al. [12] and Hafner, Anteby, Pratt et al. [14] reported significant increase in the III–V and I–V interwave intervals for several OM groups (separated by clinical state and history of treatment) compared with a control group. Persistence of delayed waves has been thought to be a reflection of the slowly recovering system than structural damage per se [9].

Delayed wave III and V, and prolonged interpeak intervals I–III and III–V are the most common findings reported in past research on children with early onset OM. However, there seems to be very little common in the operational definition of early onset OM in the studies referred to above. Early OM was OM occurring before 18 months in Gunnarson and Finitzo [10], in infancy [9], before 12 months of age [13], before 5.8 years of age at the least [11], and before 2 years 4 months at the least in Chambers et al. [8].

There is evidence to show that major changes in brain organization take place in the first year of life though changes continue into adolescence [15]. The sensori motor region witnesses the earliest myelogenesis [16]. Waves I, II and V of auditory brainstem responses are readily discernible at birth [17], while the inter-peak intervals II–III and IV–V continue to shorten during the first 2 years of life [18]. These intervals reflect trans-synaptic transmission. Matschke, Stenzel, Plath, and Zilles [19], in a study of 39 human brains ranging in age from 29 weeks of gestation to 70 years, demonstrated that myelination takes place in the first year of life which is necessary for functional maturation. It appears that normal auditory development is dependent on adequate stimulation during this sensitive period of life. There is also evidence to say that inter-peak intervals and central conduction time of the auditory brainstem responses shorten between 3^rd ^trimester of pregnancy and first 2 years of life [20]. Maturation of nerve cells in the upper nuclei as well as myelinization of small and large fibers in the auditory pathway was the reason for the reduction in central conduction time.

Synchronized encoding of transient acoustic information at the brainstem level leads to robust processing of auditory signals at the cortical level in the normal auditory system. Dys-synchronized activity at the brainstem may result in temporally degraded responses. Degraded auditory signals will not obviously result in the accurate encoding of the temporal features of the signal at the auditory cortex. Wible, Nicol and Kraus [21] recorded ABR and LLR for speech sound/da/in children with language-based learning problems and reported a good positive correlation between the results of ABR and LLR. Prolonged duration of brainstem encoding of speech sound onset, suggesting less precise timing of generation and/or transmission at inferior colliculus, was associated with weaker cortical activity. Wible et al reported 2 distinct group of subjects in whom auditory processing was different. The first group of subjects demonstrated measures of auditory signal processing at brainstem and cortical levels that were proportionate to each other. The abnormal processing of auditory signals in these subjects at the cortical level may primarily have been a result of corrupt 'input' to the thalamo-cortical circuitry which, in turn, was perhaps because of possibly degraded processing and/or transmission at the lateral lemniscus and/or inferior colliculus. A second group of children showed degraded processing at the brainstem level, but robust processing of signals at the cortical level. However, as changes in LLRs are determined, among other factors, by the integrity of underlying neural substrates at the peripheral, brainstem, and cortical levels, it is logical to say that LLRs are influenced also by the maturation and/or pathological status of the lower-level auditory processors.

Thus, cortical potentials are reported to be more sensitive than brainstem potentials in detecting subtle auditory processing deficits [22,23]. However, there are no studies which have recorded cortical potentials in children to study the effects of early onset OM. As there is some evidence to suggest that auditory processing is affected at the level of brainstem as a consequence of OM, it can be assumed that auditory cortex receives abnormal input from the brainstem which, in turn, results in abnormal auditory processing at the cortical level also. Such effects would be more pronounced if OM, and thus the reduced auditory input, occurs before 2 years of chronological age as auditory brainstem and cortical structures show greater development in the first year of a child's life.

Therefore, the purpose of this study was to determine the effect of early onset OM (occurring in the first year of life) on auditory brainstem and cortical potentials in an Indian population. A second purpose was to see the persisting nature of the effects of early onset OM. The high probability of OM, in Indian population, as a cause of conductive hearing loss [5,6] necessitates such studies in the Indian context.

## Methods

### Participants

Thirty children aged 3.1 to 5.6 years and who had OM between 6 and 12 months of their chronological age were included in the study. The selected children did not have any attacks of OM after they crossed 1 year of age. Some of the characteristics of children in the experimental groups are shown in Table 1. Children thus selected into the study were sub grouped on the basis of age to form three experimental groups (10 children in each group) as follows:

**Table 1:** Some characteristics of children in the experimental group

	**Characteristics**	**No. of children**
**Gender**	Males	15
	Females	15
**Number of episodes of otitis media**	Single	12
	Multiple	18
**Duration of otitis media**	< 1 month	12
	3–6 months	18
**Ear affected**	Unilateral	04
	Bilateral	26

a) Children in the age range of 3.1 to 3.6 years. The interval between OM and the time of testing was 2 to 3 years.

b) Children in the age range of 4.1 to 4.6 years. The interval between OM and the time of testing was 3 to 4 years.

c) Children in the age range of 5.1 to 5.6 years. The interval between OM and the time of testing was 4 to 5 years.

Grouping according to age was necessary to analyze the persistence of the effects of early onset OM, if any, on auditory processing at brainstem and cortical levels. Subjects included in the experimental group had normal results on otoscopic examination, pure tone audiometry testing (Orbiter 922 diagnostic audiometer) as well as immittance evaluation (Grason Staddler Inc. Tympstar). Most of the children in the experimental group had A type tympanogram, and normal acoustic reflexes. Six of the 30 children, 3 in the 3-year age group and 3 in the 4-year age group, had no acoustic reflexes. These children were also included in the study as their hearing thresholds were the same as that of children with normal acoustic reflexes. Results of otoscopic examination and audiological evaluations are shown in Table 2. Subjects included in the study had all been in the register of either All India Institute of Speech or Hearing, Mysore or the district hospital of the city of Mysore. Information on the early episodes of OM (between 6 and 12 months of age) was obtained from the records maintained at the institute or hospital or by family doctors. All the children belonged to the lower socio-economic strata of the society. Parents of these children had an annual income of around $ 1750 and had less than 10 years of scholastic education. Children in the experimental group were attending nursery classes or play homes in the area in which they were living.

**Table 2:** Results of otoscopic examination and audiological evaluation

		**Otoscopic examination**		**Pure tone average (dBHL)**		**Tympanogram**	
		
**Group**	**Age**	Right Ear	Left Ear	Right Ear	Left Ear	Right Ear	Left Ear
**Control**	3 yrs	Normal	Normal	12.66	13.18	A	A
	4 yrs	Normal	Normal	11.84	12.18	A	A
	5 yrs	Normal	Normal	11.74	11.94	A	A
**Experimental**	3 yrs	Normal	Normal	12.93	12.53	A	A
	4 yrs	Normal	Normal	13.66	13.18	A	A
	5 yrs	Normal	Normal	11.22	12.28	A	A

Note. Pure tone average was average of thresholds at 500 Hz, 1 kHz, 2 kHz and 4 kHz.

There were 3 control groups with 10 normal children in each group. The children in the control groups were matched for age, gender and socioeconomic status with those in the experimental groups. It was made sure that the children included in the control groups did not have a history of OM, or any other middle ear problem, by checking medical reports (if available with the family doctor), or parental reports, or by an otoscopic examination. The children in the control group underwent the same tests as children in the experimental group and had essentially normal results on otoscopic examination, and pure tone as well as impedance audiometric testing. Besides, it was ensured that none of the children included in the study had any auditory processing disorder as judged from their performance on a standard checklist for auditory processing disorder developed by Yathiraj & Mascarenhas [24].

Children in both the groups were native speakers of Kannada (a Dravidian language spoken by about 55 million people primarily in the state of Karnataka in Southern India) and belonged to the same geographical location (Mysore and the surrounding districts of Mysore). All children included in the study were attending nursery or play homes in their respective residential areas. Children were included in the study only after a written consent from one of the parents which was obtained after the parents were explained the purpose of the study. An ethics committee of the All India Institute of Speech and Hearing, headed by a retired justice of the High court of Karnataka has approved the research from the ethical angle in 2004.

### Test procedure

The protocol included recording ABRs as well as LLRs for clicks. The subjects were seated in a comfortable, relaxed position while being tested. As LLRs are reported to be affected by the state of arousal, subjects were encouraged not to sleep. A cartoon movie of the child's interest was played to ensure that the children did not sleep. The electrode sites were cleaned using Neoprep cleaning gel. Recording was through silver chloride disc electrodes. All recordings were made only after ensuring low skin impedance. Protocol for recording evoked potentials is given in Table 3. The responses were recorded for right and left ear separately.

**Table 3:** Test protocol for recording auditory brainstem responses and late latency responses

**Parameter**	**Auditory brainstem responses**	**Late latency responses**
Stimuli	Clicks	Clicks
Stimulus intensity	70 dBnHL	70 dBnHL
Transducer	TDH 39P headphones	TDH 39P headphones
Repetition rate	11.1/s	1.1/s
Stimulus polarity	Rarefaction	Rarefaction
Number of sweeps	1500	500
Filter setting	30–3000 Hz	1–30 Hz
Analysis window	-10 ms to +25 ms	-50 ms to +350 ms
Electrode montage	Vertical montage **Positive **– Cz **Negative **– M_1_, M_2_ **Ground **– Nasion	Vertical montage **Positive **– Cz, **Negative **– M_1_/M_2_ **Ground **– Nasion
Electrode impedance	< 5 kOhms	< 5 kOhms

### Data analysis

**Figure 1:**
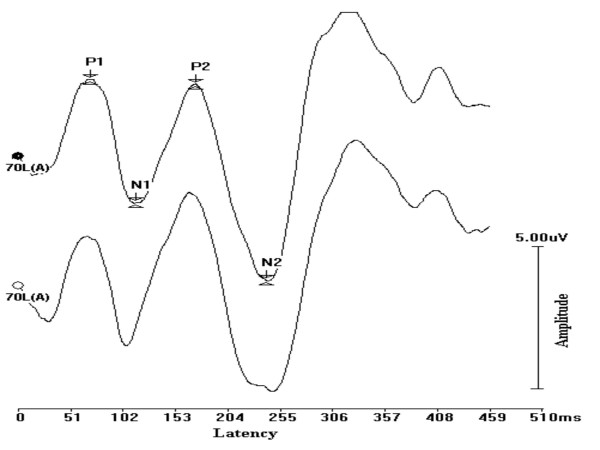
A representative late latency response recording depicting a good replicability.

Absolute peak latency, inter-peak intervals, inter aural intervals, peak amplitude and V/I amplitude ratio of ABRs were measured for each child. The ABRs and LLRs were checked for replicability by recording twice using the same protocol. Only replicable waves were considered for analysis. A representative replicable LLR recording is shown in Figure 1. Prior to analysis of individual LLR waves, grand averages of the waves were computed. This was done separately for children of different age groups. The LLR in individual subjects were identified and measured with reference to the latency range in the grand average. Wave latency and amplitudes of P1, N1, P2 and N2 were measured for each individual wave. Average baseline electrical activity was calculated for each individual recording. Amplitude, measured in the post stimulus electrical activity, was corrected with reference to the average baseline amplitude of that particular individual recording. The waves identified and the parameters measured reflect 100% agreement between the first author of the study and two other audiologists working in the area of auditory evoked potentials (with more than 10 years experience in the field).

## Results

Prior to comparison of the results of the control and the experimental groups, the responses of the two ears were compared in each of the subject (experimental and control) and age groups, using paired t test. Results showed no significant difference between the 2 ears in any of the subject groups, or in any of the age groups, either for ABR or LLR, or for any of the response parameters (latency or amplitude or interwave interval, or amplitude ratio). Therefore, data from the 2 ears were combined for further statistical analysis. The right-left combined data was normally distributed as revealed by Kolmogorov-Smirnov test of normality.

### Effect on ABRs

Independent *t *test was used to compare the results of ABR between children with and with out early onset OM. The results of all the statistical analysis, with reference to ABRs, are given in Tables 4 to 6. The major results are summarized below:

**Table 4:** Mean latencies (in ms) of click evoked auditory brainstem responses, standard deviations (SD) and the results of *t *test

**Age**	**Parameter**	**Group**	**Mean**	**SD**	***t***	***p***
3 years	Wave I	Control	1.86	0.16	*1.502*	*>0.05*
		Experimental	1.76	0.24		
	Wave III	Control	3.78	0.10	*1.836*	*>0.05*
		Experimental	3.84	0.13		
	Wave V	Control	5.67	0.15	*1.887*	*>0.05*
		Experimental	5.77	0.21		
4 years	Wave I	Control	1.80	0.10	*0.611*	*>0.05*
		Experimental	1.83	0.13		
	Wave III	Control	3.80	0.12	*1.265*	*>0.05*
		Experimental	3.86	0.15		
	Wave V	Control	5.68	0.16	*1.928*	*>0.05*
		Experimental	5.79	0.20		
5 years	Wave I	Control	1.80	0.19	*0.104*	*>0.05*
		Experimental	1.81	0.11		
	Wave III	Control	3.77	0.20	*1.282*	*>0.05*
		Experimental	3.71	0.12		
	Wave V	Control	5.68	0.25	*1.086*	*>0.05*
		Experimental	5.61	0.13		

**Table 5:** Mean inter-wave intervals and inter-aural intervals (in ms) of click evoked auditory brainstem responses, standard deviations (SD) and the results of *t *test

**Age**	**Parameter**	**Group**	**Mean**	**SD**	***t***	***p***
3 years	I–III interval	Control	1.92	0.09	*3.333*	*<0.01*
		Experimental	2.04	0.12		
	III–V interval	Control	1.89	0.09	*1.047*	*>0.05*
		Experimental	1.93	0.15		
	I–V interval	Control	3.81	0.13	*3.102*	*<0.01*
		Experimental	3.97	0.18		
	Inter-aural interval	Control	0.13	0.15	*0.230*	*>0.05*
		Experimental	0.22	0.15		
4 years	I–III interval	Control	2.00	0.99	*0.923*	*>0.05*
		Experimental	2.03	0.12		
	III–V interval	Control	1.88	0.13	*1.362*	*>0.05*
		Experimental	1.93	0.13		
	I–V interval	Control	3.88	0.16	*1.553*	*>0.05*
		Experimental	3.96	0.19		
	Inter-aural interval	Control	0.13	0.12	*0.891*	*>0.05*
		Experimental	0.08	0.07		
5 years	I–III interval	Control	1.90	0.11	*2.027*	*>0.05*
		Experimental	1.97	0.11		
	III–V interval	Control	1.91	0.11	*0.043*	*>0.05*
		Experimental	1.90	0.11		
	I–V interval	Control	3.88	0.17	*1.554*	*>0.05*
		Experimental	3.80	0.12		
	Inter-aural interval	Control	0.13	0.14	*0.426*	*>0.05*
		Experimental	0.11	0.06		

**Table 6:** Mean amplitudes (in μV) of click evoked auditory brainstem responses, standard deviations (SD) and the results of t test

**Age**	**Parameter**	**Group**	**Mean**	**SD**	***t***	***p***
3 years	Wave I	Control	0.41	0.13	*2.751*	*<0.01*
		Experimental	0.30	0.13		
	Wave III	Control	0.30	0.10	*2.619*	*<0.05*
		Experimental	0.20	0.14		
	Wave V	Control	0.59	0.17	*1.986*	*>0.05*
		Experimental	0.50	0.17		
	V/I amplitude ratio	Control	1.61	0.51	*0.131*	*>0.05*
		Experimental	2.08	1.25		
4 years	Wave I	Control	0.56	0.32	*0.409*	*>0.05*
		Experimental	0.31	0.21		
	Wave III	Control	0.43	0.17	*1.121*	*>0.05*
		Experimental	0.30	0.17		
	Wave V	Control	0.54	0.15	*0.516*	*>0.05*
		Experimental	0.50	0.26		
	V/I amplitude ratio	Control	1.77	2.25	*0.769*	*>0.05*
		Experimental	1.85	0.95		
5 years	Wave I	Control	0.29	0.16	*0.044*	*>0.05*
		Experimental	0.31	0.18		
	Wave III	Control	0.26	0.14	*0.165*	*>0.05*
		Experimental	0.34	0.30		
	Wave V	Control	0.65	0.22	*0.609*	*>0.05*
		Experimental	0.49	0.22		
	V/I amplitude ratio	Control	2.38	1.99	*0.326*	*>0.05*
		Experimental	2.88	2.13		

a) There was no significant difference between the control and the experimental groups in the mean absolute latencies of ABR in any of the three age groups (Table 4).

b) The mean I–III and I–V intervals were significantly prolonged in 3-year old children with early onset OM. This effect was not seen in children aged 4.1 years and more (Table 5).

c) The mean amplitudes of wave I and III were significantly lower in 3-year old experimental children compared to normal children of that age. Again, this effect was not seen in older children (4.1 years and above) (Table 6). Children with early onset OM, aged 4 years and above, did not differ from normal children with respect to any of the amplitude parameters.

An analysis of ABR latencies of only children who had normal acoustic reflexes was made. Results are not given here, but are available with the authors. The results are not different from that when children with no reflexes are considered.

### Effect on LLRs

**Figure 2:**
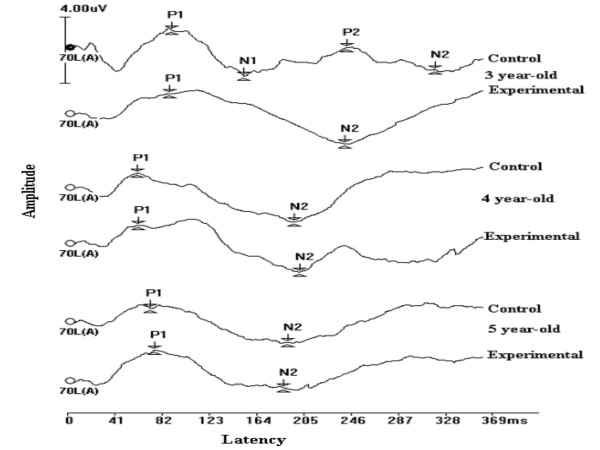
**Grand average late latency response waves**. Note. Clear N1 and P2 were not evident in the grand average late latency responses, but these two peaks were clearly seen in most of the individual recordings.

The results of all the measurements made, and statistical analysis, with reference to LLRs, are given in Tables 5 and 6. The major results are summarized below:

a) The mean latencies of P1, N1, P2 and N2 were significantly shorter in children with early onset OM compared to normal children. However, shorter latencies were noted only with 3-year old children (Table 7 & Figure 2).

**Table 7:** Mean latencies (in ms) of click evoked late latency responses, standard deviations (SD) and the results of *t *test

**Age**	**Parameter**	**Group**	**Mean**	**SD**	***t***	***p***
3 years	P1	Control	95.40	16.98	*3.013*	*<0.01*
		Experimental	81.30	12.22		
	N1	Control	168.40	32.10	*3.908*	*<0.01*
		Experimental	134.60	21.57		
	P2	Control	235.90	36.67	*5.008*	*<0.01*
		Experimental	183.55	28.98		
	N2	Control	296.80	38.38	*3.695*	*<0.01*
		Experimental	256.85	29.40		
4 years	P1	Control	79.38	16.40	*0.893*	*>0.05*
		Experimental	84.05	17.69		
	N1	Control	147.61	36.61	*0.152*	*>0.05*
		Experimental	149.20	27.40		
	P2	Control	205.55	49.03	*0.185*	*>0.05*
		Experimental	208.45	47.14		
	N2	Control	259.27	44.72	*0.699*	*>0.05*
		Experimental	270.30	51.64		
5 years	P1	Control	70.40	13.70	*0.580*	*>0.05*
		Experimental	72.95	14.11		
	N1	Control	120.68	31.36	*0.367*	*>0.05*
		Experimental	123.95	23.91		
	P2	Control	154.30	36.84	*0.840*	*>0.05*
		Experimental	163.10	28.98		
	N2	Control	217.90	34.87	*0.683*	*>0.05*
		Experimental	211.80	19.46		

b) The mean amplitude of LLR was not significantly different between children with and without early onset OM in any age group (Table 8).

**Table 8:** Mean amplitudes of click evoked late latency responses (in μV), standard deviations (SD) and the results of t test

**Age**	**Parameter**	**Group**	**Mean**	**SD**	***t***	***P***
3 years	P1	Control	2.10	2.51	*0.520*	*>0.05*
		Experimental	1.64	3.02		
	N1	Control	-1.83	3.01	*0.869*	*>0.05*
		Experimental	-1.05	2.83		
	P2	Control	2.15	1.88	*1.404*	*>0.05*
		Experimental	1.04	3.00		
	N2	Control	-2.56	2.91	*1.129*	*>0.05*
		Experimental	-3.65	3.22		
4 years	P1	Control	0.94	1.10	*0.799*	*>0.05*
		Experimental	1.46	2.54		
	N1	Control	-2.44	1.52	*2.121*	*>0.05*
		Experimental	-1.35	1.92		
	P2	Control	0.49	1.48	*1.579*	*>0.05*
		Experimental	1.33	1.76		
	N2	Control	-1.89	1.96	*0.953*	*>0.05*
		Experimental	-2.63	2.69		
5 years	P1	Control	1.26	0.88	*1.794*	*>0.05*
		Experimental	1.74	0.78		
	N1	Control	-0.94	1.51	*0.559*	*>0.05*
		Experimental	-1.19	1.38		
	P2	Control	0.58	1.91	*2.130*	*>0.05*
		Experimental	-0.53	1.95		
	N2	Control	-2.47	1.46	*0.805*	*>0.05*
		Experimental	-2.13	1.26		

### Correlation between ABR and LLR results

**Figure 3:**
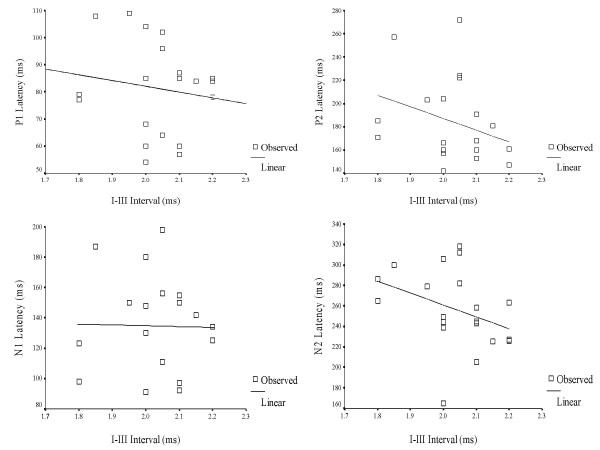
A scatter plot of the relationship between I–III intervals of auditory brainstem responses and latency of late latency responses in 3 year-old children.

**Figure 4:**
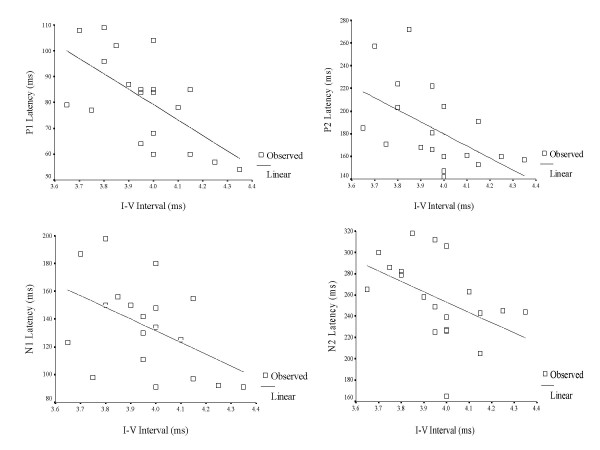
Scatter plot showing the relationship between I–V intervals of auditory brainstem responses and latency of late latency responses in 3 year-old children.

**Figure 5:**
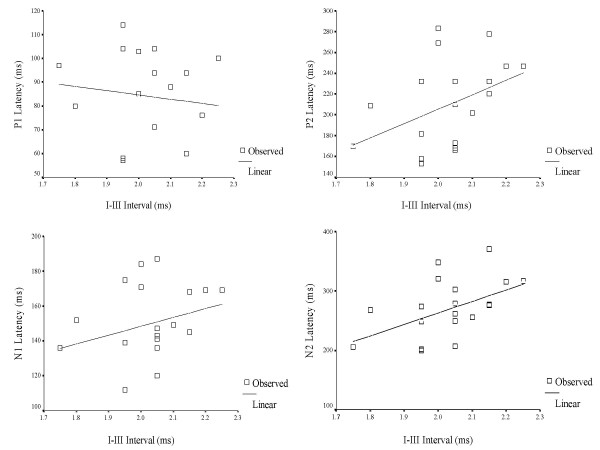
Scatter plot showing the relationship between I–III intervals of auditory brainstem responses and latency of late latency responses in 4 year-old children.

**Figure 6:**
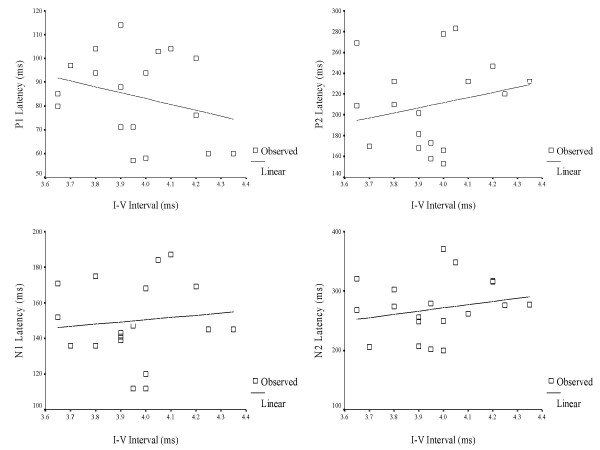
Scatter plot showing the relationship between I–V intervals of auditory brainstem responses and latency of late latency responses in 4 year-old children.

**Figure 7:**
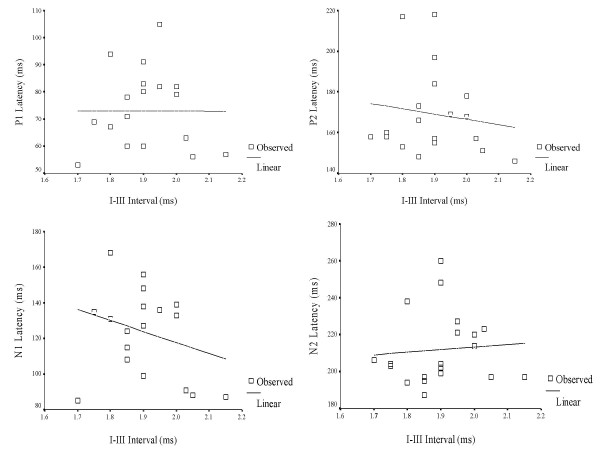
Scatter plot showing the relationship between I–III intervals of auditory brainstem responses and latency of late latency responses in 5 year-old children.

**Figure 8:**
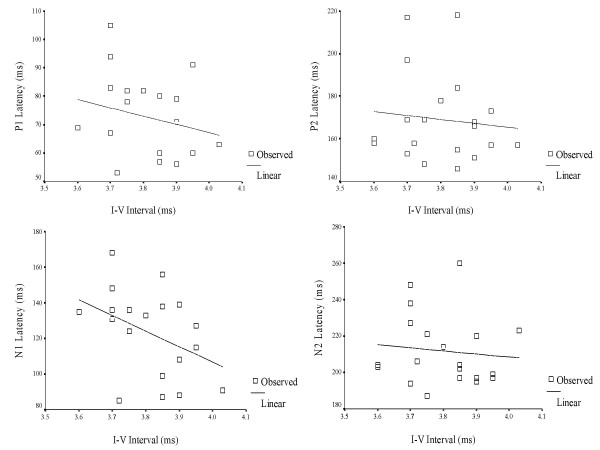
Scatter plot showing the relationship between I–V intervals of auditory brainstem responses and latency of late latency responses in 5 year-old children.

A Pearson's Product Moment correlation was computed between interpeak intervals (I – III & I – V) of ABRs and latency of LLR waves (P1, N1, P2 & N2) for 3-year old children in the experimental group (Table 9). The purpose was to understand the relationship, or bearing that a significantly longer interval (I–III & I–V) has on the timely occurrence of P1, N1, P2 and N2. In other words, whether longer interwave intervals (ABR) would, in turn, lead to delayed onset of LLRs. The results are depicted in Figures 3, 4, 5, 6, 7 and 8.

**Table 9:** Correlation coefficients, and their significance, for the relationship between interwave intervals of auditory brainstem responses and latency of late latency responses in 3 year-old children

**Parameter**	***r***	***p***
I–III interval & P1	-0.15	>0.05
I–III interval & N1	-0.02	>0.05
I–III interval & P2	-0.32	>0.05
I–III interval & N2	-0.36	>0.05
I–V interval & P1	-0.63	<0.01
I–V interval & N1	-0.47	<0.05
I–V interval & P2	-0.52	<0.05
I–V interval & N2	-0.45	<0.05

## Discussion

The results of the present study indicated that early onset OM and the consequent reduced auditory input influences auditory processing both at the lower brainstem and cortical levels in subsequent years. The results of the present study on ABR are only in partial agreement with results reported in the past. While past research has reported prolonged latencies of wave III and V [[13], among others], and increased III–V and I–V interwave intervals [[14], among others], the present study found only an increase in I–III and I–V interpeak intervals in 3-year old children who had OM in their first year of life. Any comparison of the results of the present study with that of past research, however, should take into consideration the difference in the method of the studies particularly in subject selection and characterization of 'early onset OM'.

There is no ready explanation for the results obtained in the present study, in particular the increased I–III and I–V interwave interval. An inspection of the mean absolute latencies shows that there was a relative decrease in the latency of wave I coupled with an increase in the latency of wave V in 3-year old children with early onset OM resulting in a significant prolongation of I–V interval. Physiologically, prolonged interwave intervals reflect slowing down of the central conduction time in lower brainstem.

The mean amplitude of wave I and III was significantly lower in children with OM compared to normal children, but only in the 3 year age group. Physiologically, either a decrease in the number of nerve fibers firing in response to auditory stimulus or the fibres not firing in synchrony can result in decreased amplitude. Based on the results of the present study on latency and amplitude, it is suggested that it is the auditory nerve and cochlear nuclei that are more susceptible to changes following early onset OM, but this is something to be investigated through morphological studies.

LLR peaks occurred earlier in children with early onset OM than in normal children. Again, the results were significant only in the case of 3 year old children. This was true for all 4 waves of LLR. There are no studies which have studied cortical potentials in children with early onset OM, and therefore, no comparisons are possible. However, the results of the present study do not agree with results from studies which have investigated children with auditory processing deficits [25-27]. It had been hypothesized in the present study that abnormal auditory processing at the level of brainstem may lead to deviant signal processing at the cortical level. But, the results of correlation between I–III and I–V intervals with the latency of LLRs showed an inverse relationship between the results of ABR and LLR, and the correlation was significant only for the relationship between I–V interval and LLRs. In other words, children who had prolonged conduction time showed earlier LLR and vice versa. This could be due to the phenomena of central gain in which cortical structures (increased cortical excitability) show compensatory changes when there is abnormality in the lower structures [28]. The poor morphology of LLRs (Figure 1, grand average of LLRs) is a further testimony of a 'different' type of auditory processing at the cortical level as a sequel of early onset OM.

The effect of early onset OM on ABRs (prolongation of interpeak intervals I–III and I–V, decreased peak amplitude of wave I and III), and LLRs (decreased latency of P1, N1, P2 & N2) were statistically significant only for children aged 3.1 to 3.6 years (interval between onset of OM and testing was 2 to 3 years). These effects were not observed in children aged 4.1 years and more. These results on ABR are not in agreement with those reported by Folsom et al [13], Lenhardt et al [9] or Gunnarson and Finitzo [10]. However, as has been repeatedly mentioned, the present study and those quoted above differ in their method, particularly subject selection. Also, the subjects of Gunnarson and Finitzo [10] seem to have had more severe OM while the two subjects of Lenhardt et al [9] had persistent OM since infancy. On the other hand, the subjects of the present study had only one or, at the most, two attacks, and in most cases, OM lasted for less than 3 months. Therefore, whether the severity of OM or the duration of OM are factors to be accounted while describing the long standing effects of OM needs to be investigated.

However, making a statement to the effect that the negative effects of early onset OM on ABRs and LLRs persist till the children are 3 years old and disappear thereafter may not be logical. Such an interpretation is not appropriate based on the results of a cross sectional approach because there is no way to say that subjects aged 4.1 years and more in the present study experienced negative effects of early onset OM (prolonged ABR interwave intervals and early LLRs) when they were 3.6 years or less. This calls for longitudinal studies on the topic.

An interesting observation from this study was that the early latency of LLRs seen in children aged 3.1 to 3.6 years with OM was not evident in children aged 4 years and more. Subject to the limitations of a cross sectional approach to problems of this nature, it can be said that compensatory mechanism at the cortical level is indeed a true phenomenon because once the normal conduction time was restored (4.1 years and more), LLRs occurred at expected levels of latency.

## Competing interests

The author(s) declare that they have no competing interests.

## Authors' contributions

Both the authors have made substantial contribution to conception and design, or acquisition of data, or analysis and interpretation of data, have been involved in drafting the manuscript or revising it critically for important intellectual content and have given final approval of the version to be published.

## References

[B1] Knudsen EI (1985). Experiences alters the spatial tuning of auditory units in the optic tectum during a sensitive period in the barn owl. J Neurosci.

[B2] Sandeep M, Jayaram M Effect of early otitis media on speech perception. Under editorial process.

[B3] WHO (2004). Chronic suppurative otitis media. Burden of illness and management options. http://www.who.int/child-adolescent-health/New_Publications/CHILD_HEALTH/ISBN_92_4_159158_7.pdf.

[B4] Teele D, Klein J, Rosner B (1980). Epidemiology of otitis media in children. Ann Otorhinolaryngol.

[B5] Jayaram M (2007). Assessment of etiological factors of conductive hearing loss.

[B6] Parsram K, Jalvi R (2007). Assessment of etiological factors for conductive hearing Impairment in general population in Mumbai (All age groups).

[B7] Webster DB, Webster M (1979). Effects of neonatal conductive hearing loss on brainstem auditory nuclei. Ann Otol Rhinol Laryngol.

[B8] Chambers RD, Rowan LE, Matthies ML, Novak MA (1989). Auditory Brain-stem Responses in Children with Previous Otitis Media. Arch Otolaryngol Head Neck Surg.

[B9] Lenhardt ML, Shaia FT, Abedi E (1985). Brain-stem evoked response waveform variation associated with recurrent otitis media. Arch Otolaryngol.

[B10] Gunnarson AD, Finitzo T (1991). Conductive hearing loss during infancy: effects on later auditory brainstem physiology. J Speech Lang Hear Res.

[B11] Hall JW, Grose JH (1993). The effect of otitis media with effusion on the masking-level difference and the auditory brainstem reponse. J Speech Hear Res.

[B12] Anteby I, Hafner H, Pratt H, Uri N (1986). Auditory brainstem evoked potentials in evaluating the central effects of middle ear effusion. Int J Pediatr Otorhinolaryngol.

[B13] Folsom RC, Weber BA, Thompson G (1983). Auditory brainstem responses and children with early recurrent middle ear disease. Ann Otorhinolaryngol.

[B14] Hafner H, Anteby I, Pratt H, Golsher M, Shenhav R, Joachims HZ (1986). Auditory brainstem evoked potentials in evaluating the efficacy of surgical ventilation of the middle ear. Int J Pediatr Otorhinolaryngol.

[B15] Huttenlocher P (1979). Synaptic density in human frontal cortex – Developmental changes and effects of aging. Brain Res.

[B16] Vaughan HG, Kurtzberg D, Gunnar MR, Nelson CA (1992). Electrophysiologic indices of human brain maturation and cognitive development. Developmental Behavioral Neuroscience.

[B17] Picton TW, Taylor MJ, Durieux-Smith A, Aminoff M (1992). Brainstem auditory evoked potentials in pediatrics. Electrodiagnosis in Clinical Medicine.

[B18] Ponton CW, Moore JK, Eggermont JJ (1996). ABR generation by parallel pathways: Differential maturation of axonal conduction time and synaptic transmission. Ear Hear.

[B19] Matschke RG, Stenzel C, Plath P, Zilles K (1994). Maturational aspects of the human auditory pathway: anatomical and electrophysiological findings. ORL J Otorhinolaryngol Relat Spec.

[B20] Inagaki M, Tomita Y, Takashima S, Ohtani K, Andoh G, Takeshita K (1987). Functional and morphometrical maturation of the brainstem auditory pathway. Brain Dev.

[B21] Wible B, Nicol T, Kraus N (2005). Correlation between brainstem and cortical auditory processes in normal and language-impaired children. Brain.

[B22] Jirsa RE, Clontz BC (1990). Long latency auditory event related potentials from children with auditory processing disorders. Ear Hear.

[B23] Jerger J, Musiek F (2000). Report of the consensus conference on the diagnosis of auditory processing disorders in school-aged children. J Am Acad Audiol.

[B24] Yathiraj A, Mascarenhas K (2003). Effect of auditory stimulation in central auditory processing in children with CAPD.

[B25] Scatterfield JH, Schell AM, Backs RW, Hidaka KC (1984). Cross sectional and longitudinal study of age effects of electrophysiological measures in hyperactive and normal children. Biol Psychiatry.

[B26] Leppanen T, Lyytinen H (1997). Auditory event related potentials in the study of developmental language related disorders. Audiol Neurootol.

[B27] Arehole S (1995). A preliminary study of the relationship between long latency response and learning disorder. Br J Audiol.

[B28] Salvi RJ, Wang J, Ding D, Stecker N, Arnold S (1999). Auditory deprivation of central auditory system resulting from selective inner hair cell loss: Animal model of auditory neuropathy. Scand Audiol.

